# Comparison of Shear Bond Strength of Three Types of Adhesive Materials Used in the Restoration of Permanent Molars after Treatment with Silver Diamine Fluoride: An In Vitro Study

**DOI:** 10.3390/ma16216831

**Published:** 2023-10-24

**Authors:** Mannaa K. Aldowsari, Fatimah Alfawzan, Alanoud Alhaidari, Nada Alhogail, Reema Alshargi, Saad Bin Saleh, Ayman M. Sulimany, Mohammed Alturki

**Affiliations:** 1 Department of Pediatric Dentistry and Orthodontics, College of Dentistry, King Saud University, Riyadh 12372, Saudi Arabia; 2 College of Dentistry, King Saud University, Riyadh 12372, Saudi Arabia; 3 Department of Restorative Dental Science, College of Dentistry, King Saud University, Riyadh 12372, Saudi Arabia; mohaalturki@ksu.edu.sa

**Keywords:** caries, composite, dental, SDF

## Abstract

Background: Permanent blackish discoloration of the tooth structure post application of silver diamine fluoride (SDF) is one of its drawbacks. Several restorative materials have been used to restore and mask the blackish discoloration of SDF-treated teeth. Recently, a new self-adhesive material has been introduced and is marketed as an all-in-one etchant, adhesive, and restorative material indicated for use in all clinical situations. This study aimed to assess the shear bond strength of the new self-adhesive restorative material and compare it with adhesive restorative materials- resin-based composite and resin-modified glass ionomer cement to dentin of extracted permanent teeth treated with 38% SDF. Methods: Thirty-nine caries-free extracted teeth (*n* = 39) were grouped into three groups. Following 38% SDF application, the specimens were loaded with resin-based (Group I), the new self-adhesive restorative material (SDR) Surefil (Group II), and resin-modified glass ionomer cement (RMGIC) (Group III). Shear bond strength (SBS) was calculated, and failure modes were evaluated using the universal testing device (3) Results: The composite showed the highest bond strength, followed by Group II while Group III had the lowest bond strength of all tested materials. Regarding failure type, the composite showed 100% adhesive failure, while Group III and Group II showed mostly adhesive failure with some combination. (4) Conclusions: RBC had a significantly stronger SBS to demineralized dentin surfaces of permanent molar teeth treated with SDF when compared to SDR Surefil and RMGIC.

## 1. Introduction

Dental caries is a dynamic, biofilm-mediated disease that causes phasic demineralization and remineralization of dental hard tissues. It results from complex interactions between acid-producing cariogenic bacteria, substrates like carbohydrates, and other host factors such as saliva and teeth [1]. Permanent and primary dentitions are susceptible to caries throughout life, which can affect the tooth’s crown and, in the long term, exposed root surfaces [2]. 

Dental caries affecting children under six years of age are called early childhood caries (ECC). ECC spreads quickly and can cause children to experience extreme pain, abscesses, swelling, fever, and psychological disorders [3]. Two practical approaches for ECC prevention before the onset of cavitation are the use of fluoride varnishes containing 5% sodium fluoride (NaF) and fluoridated toothpaste [4]. In cases of cavity formation in ECC, removal of the infected tooth tissue and restoration are recommended. 

SDF, a combination of silver nitrate and fluoride- in a 38% concentration has recently been used (off-label) as a caries-arresting agent [5]. Silver nitrate and fluoride work together to create SDF, thereby acting as both an anti-microbial as well as a remineralizing agent [6]. Silver phosphate and calcium fluoride are the products of the SDF’s reaction with hydroxyapatite, and they operate as a reservoir for fluoride and phosphate ions that encourage remineralization. The silver ions enter the lesions (up to 30 microns into the enamel, up to 300 microns into the dentin, and up to 2 mm in a deep carious lesion) and exert their anti-bacterial effect [5,6]. 

The treatment’s simplicity makes it suitable for treating caries in young children who may have intense dental fear, uncooperative patients with special needs, or elderly patients who have difficulty adapting to traditional dental care. Its straightforward application procedures do not require injection or drilling [6]. However, due to the presence of silver compounds like silver oxide and silver phosphate, the carious lesions stain black permanently. This influences the patient’s and the parents’ acceptability of the treatment. In efforts to mask the black staining, many clinicians place adhesive white or tooth-colored restorative materials as direct restorations in the cavitated lesions after SDF application [7]. 

Recently, a new self-adhesive material restorative material (SDR) Surefil, Dentsply Sirona, Konstanz, Germany) has been introduced as an all-in-one etchant, adhesive, and restorative material for restoring primary and permanent teeth. It offers the glass ionomer’s simplicity and speed and a composite materials’ restorative longevity and stability. The main component of Surefil One is MOPOS, a modified polyacid, which has a unique structure to allow new opportunities for creating self-adhesive restorative materials. MOPOS enables the material to adhere to tooth structure and form networks, which increases the material’s mechanical strength. The addition of polymerizable groups to the polyacid base polymer, which is hydrolytically stable, is what distinguishes MOPOS from other technologies [8]. As the material is applied in one layer without needing adhesive or special retentive preparations, it makes it the ideal restorative material in clinical cases where time or cooperation aspects must be considered, as is the case with plenty of pediatric patients. 

The number of clinical and in-vitro studies to test the biological, mechanical, and optical properties of this material is currently limited. In a clinical study by Rathke et al., the newly introduced material has shown acceptable clinical results over the follow-up period of one year. The study concluded that the restorations were in clinically acceptable condition with an annual failure rate of 2%. However, color stability showed the most significant change over time [9]. The mechanical properties of SDR in comparison with three direct composite resins and two GIC materials as evaluated by Lohbauer and Belli were found to be in a range similar to the resin composites [10]. Francois et al., on studying the share bond strength (SBS) of SDR One found the highest bond strength values among the tested materials in samples without any pre-treatment with a universal adhesive followed by etching [11]. Similar results were also found by Sadeghyar et al. in an animal study measuring the SBS values of different materials with and without pre-treatment [12]. However, data regarding the effect of SDF on the bond strength of this new self-adhesive material is inconclusive. 

Therefore, the aim of this study was to assess the SBS of three different types of adhesive restorative materials, including resin-based composite (RBC), a new Self-adhesive restorative material (SDR) Surefil, and resin-modified glass ionomer cement (RMGIC) of dentin of extracted permanent teeth treated with 38% SDF. The null hypothesis was that there was no difference between the SBS of the three different materials with the dentin treated with 38% SDF.

## 2. Materials and Methods

An in vitro study was conducted to assess the SBS of three different types of adhesive restorative materials. The three materials included a resin-based composite (Neo Spectra ST LV, Dentsply Sirona, Charlotte, NC, USA) (RBC), a new self-adhesive restorative material (Dentsply Sirona, Konstanz, Germany) (SDR) Surefil and resin-modified glass ionomer cement (RMGIC, Fuji IX, GC Corporation, Tokyo, Japan) (RMGIC). The chemical composition of the investigated materials is presented in Table 1. 

### 2.1. Specimens and Sampling Technique

A power analysis was conducted to calculate the sample size. Based on previously available literature, using a package (pwr) in R software (R package version 1.3-0.) with a 95% confidence interval and 80% power of the study, the sample size was determined. Ethical clearance was obtained from the Institutional Review Board of the university. 39 permanent sound teeth indicated for orthodontic extraction were collected from dental clinics in Riyadh, Saudi Arabia after a written informed consent from the patients. The inclusion criterion was sound teeth. The exclusion criteria were teeth with restoration or caries. To remove debris and calculus, blood, and plaque, the teeth were thoroughly cleaned with an ultrasonic scaler and then placed in freshly prepared 0.5% chloramine-T solution and stored at 4–7 °C until further use. The sample was randomly split into three groups (*n* = 13) using a simple random sampling technique as follows: the first group (I) was loaded with RBC, the second group (II) was loaded with SDR Surefil and the third group (III) was loaded with RMGIC.

### 2.2. Specimens Preparation

The teeth were mounted in round metal molds in cold-cure clear acrylic. A slow-speed cutting machine (IsoMet, Buehler, Plymouth, MN, USA) was used to remove the occlusal enamel of the teeth specimens. The complete removal of the enamel was ensured by examining the dentin surfaces of the specimens under a stereomicroscope (SM80, Swift microscope, Carlsbad, CA, USA). The specimens were demineralized for seven days to simulate caries with pH adjusted to 5.0 (acidic) at 37 °C.

### 2.3. SDF Treatments

The occlusal surface of the demineralized dentin specimens was treated uniformly with one drop of 38% SDF (Advantage Arrest, Elevate Oral Care, West Palm Beach, FL, USA) using a micro brush applicator tip without a pre-etching step. After the SDF application had dried using a gentle flow of compressed air for one minute without rinsing, the dentin specimens were stored at 37 °C in distilled water for two weeks. After two weeks, the dentin specimens were loaded with assigned adhesive restorative materials as per the manufacturers’ recommendations for each adhesive material (Table 1).

After loading the specimens with assigned restorative materials, the specimens were then loaded in a thermocycling machine (Huber, SD-Mechatronik-Thermocyclerr, Berching, Germany) and subjected to 5000 cycles of thermocycling between 5 °C and 55 °C to mimic 6 months of physiological use. The dwell time in each bath was set to 30 s.

### 2.4. SBS Measurements and Failure Modes

The universal testing device evaluated the SBS (Instron 5965, Norwood, MA, USA). The specimens were mounted in a metal mold 3 mm in diameter, serving as a drive surface for a metal plunger. This plunger touched the cylindrical test material at the contact point with the dentin at right angles. The testing device moved with a defined 1 mm/min speed toward the plunger. The shear bond strength was calculated with a special software program (Blue Heal 3). Failure modes were evaluated utilizing a stereomicroscope (SM80, Swift microscope, Carlsbad, CA, USA) and classified as adhesive, cohesive, and mixed failures.

### 2.5. Statistical Analysis

The statistical analysis used was the Statistical Package for the Social Sciences (SPSS) 19.0 (v.19.0, IBM, Chicago, IL, USA). Mean and standard deviation from the recorded shear bond strength values were calculated. Analysis of variance (ANOVA) with the Tukey Post hoc test was done to compare the bond strength between the three groups. 

## 3. Results

The SBS of the three materials was tested, and the mean and standard deviation were calculated. The intergroup comparison was done using one-way ANOVA followed by Tukey HSD post hoc test (Table 2). 

**Figure 1:**
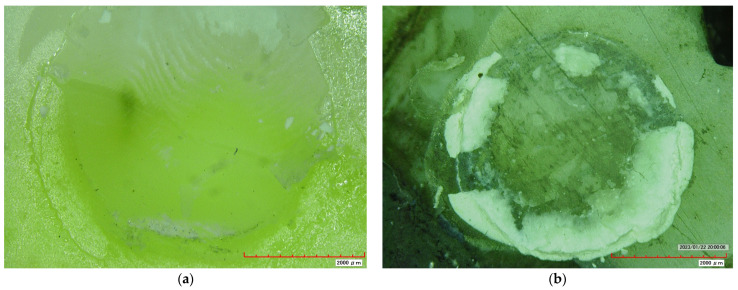
Stereomicroscope images at (×40) show the mode of failure of different experimental groups. (**a**) representative sample showing the adhesive type of failure of RBC specimen (**b**) representative sample showing the mixed type of failure of SDR surefil specimen.

RBC showed the highest bond strength, followed by SDR Surefil, while RMGIC had the lowest bond strength of all tested materials. Statistically significant results were found between the RMGIC and RBC groups and between SDR Surefil and RBC groups when SBS was calculated after pretreatment of dentin with 38% SDF. However, no statistically significant difference was found between SDR Surefil and RMGIC (Table 2). Regarding the type of adhesive failure, RBC showed 100% adhesive failure, while RMGIC and SDR Surefil showed mostly adhesive failure with some combination. No cohesive failures were noted in any of the groups (Table 3) (Figure 1).

## 4. Discussion

The aim of this in-vitro study was to evaluate the effect of pre-treatment of dentin with 38% SDF on the bond strength of three different restorative materials. In-vitro studies are often used to test the different variables of newly introduced materials and their interactions with other biomaterials in common clinical scenarios. SBS reveals the adhesive strength of the material at the restoration-tooth interface. It is at this interface that the forces of mastication, which are analogous to the shearing phenomenon, result in a complicated stress distribution during clinical weight-loading situations [13]. 

In this study, the maximum SBS was noted with the RBC, followed by SDR Surefil, and the least values were noted with RMGIC. Statistically significant results were found between the RMGIC and RBC groups and between SDR Surefil and RBC groups when SBS was calculated after pretreatment of dentin with 38% SDF. 

SDF has become a popular alternative in managing dental caries, which was approved by the US Food and Drug Administration (FDA) in 2014 as a commercial product for dental use. However, concerns have been raised about its impact on the bond strength of restorative materials to dentin [14]. Multiple in-vitro studies have been conducted to investigate SDF’s effects on the bond strength of restorative materials. Zhao et al. showed that pre-treatment with SDF did not negatively affect the adhesion of GIC to caries-affected dentine [15]. Similarly, Wu et al. showed that the bond strengths of composite to sound primary molars were not affected using 38% SDF on primary dentin [16]. In contrast, pre-treatment of sound primary dentin with SDF significantly increased the SBS between RMGIC and primary dentin as studied by Sa’ada et al. [17]. In addition, the light curing of RMGIC for 20 s may increase the SBS between SDF pre-treated primary dentin and RMGIC [18]. Fröhlich et al. in their systematic review stated that the effect of SDF on dentin bonding was material-dependent, no effect was noted on adhesion of GIC, but a significant decrease in bond strength with adhesive systems was observed [19]. In an updated systematic review published by the same authors, it was found that SDF application, followed by rinsing, does not jeopardize adhesive bond strength, and could improve the adhesion of the restorative material to caries-affected dentin [20]. The mean SBS in the present study was lower than the universal adhesive values. This could be due to the effect of pre-treatment of SDF as shown previously in a recent systematic review, which showed a significant decrease in bond strength with adhesive systems [19]. 

An ideal restorative material should have optimal biocompatibility, chemical adhesion, adequate strength, and be usable in all clinical situations. SDR Surefil was introduced by the manufacturer as a one-step bulk-fill material, eliminating the need for etching and bonding procedures, and was indicated for use in all clinical situations. It has initiators to enable both photo and chemo polymerization and a high molecular weight polymer called MOPOS by the manufacturer. This polymer is claimed to promote bonding to the tooth and create a strong composite-like structure [20]. Abuljadayel and co-workers in an invitro study, studied the effect of SDF and Chlorhexidine on the SBS of various bio-active restorative materials, including SDR Surefil [8]. Higher values were noted with the SDF-treated specimens as compared to the chlorhexidine group. However, the results of this study must be treated with caution as this study assessed immediate SBS values without any artificial aging. This is not in line with the recent recommendations which recommend placement of a restorative material two weeks after treatment with SDF to minimize black discoloration [21]. 

The RMGIC, evaluated in this study, is a commonly used material in posterior restorations and ART (atraumatic restorative treatment). There are numerous studies comparing the bonding of GIC with and without surface treatment of SDF. No solid conclusion was drawn in a systematic review by Jiang et al. based on this topic [14]. A high degree of variation of data comparing the effect of SDF application on the bond strength of dentine to adhesives and to GICs was the reason behind their findings. Ng et al. found no difference in the bond strength of glass ionomer cement to dentin lesions after SDF treatment. They noted improved retention by allowing the SDF solution to be set for one week prior to GIC placement [22]. Abdullah et al. in an in vitro study involving primary molars, have noted lower SBS for GIC, composite resin, and resin-modified bioactive resin for SDF-treated dentin as compared to the control group (sound dentine without SDF) [23]. 

Of the three restorative materials tested in this study, Neo Spectra ST is a nano-hybrid composite with a patented SphereTEC technology as claimed by the manufacturer. The fillers have a micro-granulated structure, thereby allowing them to bind more free resin than conventional fillers [24]. Our results showed that RBC exhibited the highest SBS after SDF treatment. This was probably because the etching and bonding step was performed only for this material group. It is well known that the bonding of adhesives is based on micromechanical retention and hybrid layer formation. SDF is a highly alkaline fluid that reacts with hydroxyapatite forming a silver phosphate layer, resulting in an impermeable layer and dentinal tubule obstruction, making it both a physical and chemical barrier to adhesion [25,26,27]. This interferes with resin impregnation, thereby affecting the SBS of composite resin. The application of phosphoric acid has been suggested by Koizumi et al. to remove some precipitate SDF and thereby increase the bond strength [28]. Using an etchant and delaying restoration placement positively affects the SBS of the composite.

Similarly, there is no consensus on the effect of immediate rinsing of SDF with water on the bond strength of the adhesive materials. While some authors have noted increased bond strength values after rinsing SDF-applied specimens with water [15,29], others have reported no significant changes in the same [30,31]. There was no rinsing of the specimens with water in this study, which could have possibly influenced the final results. Francois et al., had compared the differences in SBS of SDR Surefil, with or without the use of an adhesive system, and recorded higher values for specimens where an adhesive was used [11]. In the same study, when different materials SDR Surefil and Activa BioActive Restorative) were compared without the use of an adhesive, SDR Surefil demonstrated the highest SBS. They attributed this high adhesion value to the structural composition of Surefil. The functionalized polyacrylic acid of high molecular weight facilitates hybridization of the smear layer, interactions between calcium contained in dentin and carboxyl groups of MOPOS have been shown to promote adhesion between the material and the tooth structure [32]. In another study by Latta et al., similar SBS values were found between light-cured and self-cured SDR Surefil specimens [33]. However, no adhesives were used in this case. They concluded that on dentin surfaces, the self-adhesive materials including SDR Surefil generated lower SBS values than a composite resin and a universal adhesive. The results of this study are on similar lines as our study where the values of SDR Surefil were similar to RMGIC but lesser than RBC.

In terms of failure, RBC showed 100% adhesive failure. This is supported by Aldosari et al. and Atalay et al., who found adhesive failure to be the more frequent mode [34,35]. Most RMGIC and SDR Surefil samples had an adhesive failure, with some samples showing combination failure. This contrasts with Poorzandpoush K. et al., which found RMGIC to have 100% adhesive failure [36]. There were no cohesive failures with any of the samples. Instead of indicating the bonding properties, cohesive failures often indicate different mechanical properties of the materials involved or some other problems like errors in alignment of the testing assembly, or microcracks in the specimens. Scherrer et al. in their literature review have suggested avoiding cohesive failure specimens and analyzing data from specimens with adhesive failure or mixed failure with small region (<10%) only, to get more accurate results [37]. Ignoring any data may however lead to bias in the final results and should be treated with caution.

The main limitation of this study is that it is an in vitro study; it cannot mimic the in vivo conditions. However, Thermocycling was used in this study to simulate some aspects of the oral environment to overcome this limitation. Moreover, SDF is mainly used in primary teeth. However, in this study, permanent molars were used instead of primary teeth. The differences in the dentinal tubules’ structures may also be a factor affecting the action of SDF on the natural tooth and the subsequent restorative material. Another limitation could be that the bond strength of teeth affected by clinical caries may be different from artificial caries. However, in spite of these limitations, the present study’s findings can contribute to the ongoing studies that investigate the effect of pre-treatment of SDF on the physical properties of the dental materials.

The interface between the restoration and the SDF-treated tooth is exposed to diverse forces that act simultaneously in the oral cavity. Therefore, long-lasting clinical trials remain necessary to validate the laboratory observations. Only the shear bond strength was calculated in this study. Further scope of the research includes the assessment of other mechanical and optical properties of the new self-adhesive material, SDR Surefil, in SDF-treated teeth and correlating its clinical survival and success rates. Also, further studies using different restorative materials might be considered. 

## 5. Conclusions

Within the limitations of this in vitro study, the following conclusions were made:

1. RBC had a significantly stronger SBS to demineralized dentin surfaces of permanent molar teeth treated with SDF when compared to SDR Surefil and RMGIC.
2. SDR Surefil showed better properties of SBS compared with RMGIC, making it a good alternative choice to conventional restorative materials.
3. The bond strength between SDF-treated tooth and SDR Surefil found to be within the clinical acceptable limits, makes the material a good alternative choice to be used to mask the discoloration caused by SDF application. This is particularly helpful in young and un-cooperative patients since the material is marketed by the manufacturer as a self- adhesive material without the requirement of any tooth preparation procedures.

## Figures and Tables

**Table 1:** Materials used in this study and their composition and application mode.

Material, Manufacturer	Composition	Application Mode
Advantage arrest, silver diamine fluoride 38%, Elevate Oral Care, West Palm Beach, FL, USA)	Silver fluoride, ammonia, and deionized water	-Dry the surface -Apply the material using a micro brush to the tooth surface -Dry with a gentle flow of compressed air for one minute.
Best-Etch, Vista Dental designs, New York, NY, USA	Phosphoric acid 37%	-Apply to the surface of the bonding for 15 s. -Rinse with water for 5 s and dry.
Prime & Bond NT, Dentsply Sirona)	MDP Phosphate Monomer, dimethacrylate, HEMA, Vitrebond copolymer, fillers, ethanol, water, initiator, and saline	-Apply for 20 s using a microbrush. -Air dry gently for 5 s. -Cure for 10 s
Neo Spectra ST LV, Dentsply Sirona	A blend of spherical, pre-polymerized Sphere fillers (d3,50 ≈ 15 µm), non-agglomerated barium glass and ytterbium fluoride. Highly dispersed, methacrylic polysiloxane nano-particles	-Apply 2 mm thickness and light cure for 20 s
Surefil One, Dentsply Sirona, Konstanz, Germany	Aluminum-phosphor-strontium- sodium-fluoro-silicate glass, water, silicon dioxide, acrylic acid, polycarboxylic acid, ytterbium fluoride, bifunctional acrylate, self-cure initiator, pigments, camphorquinone, and stabilizer	-Activate capsule, and place in a mixer for 10 s -Apply 2 mm and light cure for 20 s.
Fuji IX, GC Corporation, Tokyo, Japan (RMGIC)	Powder: Fluro alumino silicate glass, Polyacrylic acid powder. Liquid: Polyacrylic acid Polybasic carboxylic acid	-Activate capsule, -Place in a mixer for 10 s, -Apply 2 mm then let it set for 3 min.

Abbreviations: HEMA—Hydroxyethyl methacrylate; MDP: 10-methacryloyloxydecyl dihydrogen phosphate.

**Table 2:** Comparison of the mean shear bond strength of the test materials using one-way ANOVA and between group comparison using Tukey HSD post hoc test.

Study Group	*n*	Mean in (Mpa)	Std. Dev.	*p* Value
RBS	13	21.83	2.49	<0.0001
RMGIC	13	15.70	2.06	
SDR Surefil	13	17.71	1.79	
**Between group comparison**	**Difference between means**	**95% Confidence Limits**		***p* value**
RBC vs. SDR Surefil	4.1204	2.0764	6.1645	<0.0001
RBC vs. RMGIC	6.1313	4.0872	8.1753	<0.0001
SDR Surefil vs. RMGIC	2.0108	−0.0332	4.0549	0.055

**Table 3:** Types of failure for the study groups.

Restorative Material	Adhesive *n* (%)	Cohesive *n* (%)	Combination *n* (%)	Total *n* (%)
RBC	13 (100%)	0 (0%)	0 (0%)	13 (100%)
SDR Surefil	9 (69.23%)	0 (0%)	4 (30.76)	13 (100%)
RMGIC	10 (76.92%)	0 (0%)	3 (23.07)	13 (100%)

## Data Availability

The database is available upon request from the corresponding author).
